# Analyzing Medication Adherence Patterns Among Type 2 Diabetes Patients in Thi-Qar, Iraq: A Cross-Sectional Study

**DOI:** 10.1155/jdr/6659722

**Published:** 2025-05-16

**Authors:** Adel Gassab Mohammed, Nassar Taha Yaseen, Dheyaa K. AlWaeli

**Affiliations:** ^1^Department of Medicine, Endocrine and Metabolism Division, University of Thi-Qar, College of Medicine, Thi-Qar Specialized Diabetes, Endocrine, and Metabolism Center (TDEMC), Thi-Qar Health Directorate, Nasiriyah, Thi-Qar, Iraq; ^2^Faiha Specialized Diabetes, Endocrine and Metabolism Center, University of Basrah, Basrah, Iraq

**Keywords:** cross-sectional study, glycemic control, Southern Iraq, treatment adherence, Type 2 diabetes

## Abstract

This research investigates the adherence levels to diabetes treatment among patients and explores the factors influencing adherence, glycemic control, and the occurrence of diabetes-related complications. A cross-sectional study involving 296 diabetes patients was conducted to evaluate their demographic and clinical profiles, treatment strategies, and adherence levels using the eight-item Morisky Medication Adherence Scale (MMAS-8). Statistical analyses identified variables affecting adherence and their relationships with glycemic control and complications. The study population comprised 56% men, with an average age of 49.4 years. Obesity was observed in 24.3% of participants, while the median disease duration was 5 years. The average HbA1c level was 8.4%. Microvascular complications were present in 48.6% of patients, and 18.2% experienced macrovascular complications. Most patients were prescribed oral antidiabetic medications (OAD), with 25% receiving insulin therapy. Adherence rates were suboptimal, with only 3.4% achieving good adherence, 30.4% moderate adherence, and 66.2% poor adherence. Men had higher rates of poor adherence compared to women (72.3% vs. 58.5%). Interestingly, adherence was better in patients with a longer disease duration and a higher body mass index (BMI). The study further examined adherence's impact on glycemic outcomes, finding that poor adherence strongly correlated with elevated HbA1c levels. Among individuals with HbA1c ≥ 7%, 70.5% exhibited poor adherence, whereas 40% of patients with good adherence still had suboptimal glycemic control. Conversely, among those with HbA1c < 7%, 24.5% demonstrated poor adherence compared to 40% with good adherence. Random blood sugar (RBS) levels were significantly higher in poorly adherent patients (237 mg/dL) versus those with good adherence (141 mg/dL). Although adherence was not statistically linked to complication prevalence, patients with reported adherence challenges were more prone to both microvascular and macrovascular complications. Different treatment regimens were also analyzed, revealing that sulfonylureas (SUs) were associated with poor adherence (85%), while sodium–glucose cotransporter 2 inhibitors (SGLT2i) showed better adherence rates (16.7%). Missed doses were strongly linked to poor glycemic outcomes but had a lesser impact on complication development. These findings underscore the need for individualized strategies to enhance adherence and optimize glycemic control, ultimately reducing diabetes-related complications.

## 1. Introduction

Type 2 diabetes mellitus (T2DM) is a significant global health concern, impacting a substantial portion of the adult population. Achieving and maintaining glycemic control is essential for managing T2DM effectively and minimizing the risk of complications, which heavily relies on optimal medication adherence. Despite this, nonadherence to prescribed treatments remains a widespread issue in T2DM management [1].

One key factor affecting medication adherence is diabetes-related distress, encompassing the psychological and emotional challenges associated with living with T2DM. Factors such as complex treatment regimens, fear of hypoglycemia, and lifestyle changes contribute to heightened distress, which can negatively impact patients' ability to adhere to their medication schedules [2].

This cross-sectional study titled “A Cross-Sectional Analysis of the Relationship Between Diabetes-Related Emotional Distress and Medication Adherence in Adults With Type 2 Diabetes Mellitus in Thi-Qar, Iraq” was conducted to explore the relationship between diabetes-related distress and medication adherence in adult T2DM patients. The research was carried out at the Thi-Qar Specialized Diabetes, Metabolism, and Endocrine Center (TDEMC) in Thi-Qar City, Iraq, involving 296 participants recruited between January 2023 and June 2023.

The primary aim of the study was to assess the concurrent validity of the eight-item Morisky Medication Adherence Scale (MMAS-8) by examining its relationship with glycosylated hemoglobin (HbA1c) levels in patients with T2DM. The MMAS-8, a widely recognized tool for evaluating medication adherence, focuses on factors such as forgetfulness, medication-taking behavior, and barriers to adherence [3]. This study sought to understand how adherence, as measured by the MMAS-8, impacts glycemic control in T2DM patients.

Recognizing the connection between diabetes-related distress and medication adherence is critical for healthcare providers to develop effective, tailored interventions. Existing research highlights the importance of addressing psychological factors to enhance adherence in T2DM management [4]. For instance, Kretchy et al. demonstrated a significant link between high levels of diabetes-related distress and poor medication adherence in T2DM patients [5]. Similarly, Schmitt et al.'s systematic review found that interventions targeting psychological aspects improved adherence in chronic conditions, including T2DM [6].

The results of this study contribute valuable insights into the relationship between diabetes-related distress and medication adherence, emphasizing the need for patient-centered strategies. These findings can guide healthcare professionals in designing interventions to improve adherence, enhance glycemic control, and ultimately improve the quality of life for T2DM patients.

## 2. Methodology

### 2.1. Study Design

A cross-sectional study was conducted to evaluate treatment adherence among patients with diabetes and to explore factors influencing adherence levels, glycemic control, and the development of diabetes-related complications in Thi-Qar City, Southern Iraq.

### 2.2. Participant Recruitment

Consecutive sampling was employed: all eligible patients visiting the clinic during the study period were invited until the target sample size (*n* = 296) was reached. Recruitment occurred across all clinic days (weekdays/weekends) and shifts (morning/afternoon) to ensure representativeness.

Include a brief rationale: consecutive sampling was chosen to reflect the clinic's routine population while maintaining practicality in a resource-limited setting.

To compare two independent means, the sample size was calculated using the following equation:

 $$\begin{array}{c} n=\left(\left(Z\alpha /2+Z\beta \right)2\cdot \left(SD12+SD22\right)\left(M1-M2\right)2\right)n=\left(\left(M1-M2\right)2\left(Z\alpha /2+Z\beta \right)2\cdot \left(SD12+SD22\right)\right) \end{array}$$

where *Zα*/2 is the *Z* value for alpha level (for *α* = 0.05, *Zα*/2 ≈ 1.96) and *Zβ* is the *Z* value for desired power (for power = 0.80, *Zβ* ≈ 0.84). SD1 and SD2 are the standard deviations of the two groups (assuming equal variance for simplicity). *M*1 − *M*2 is the expected difference in means. A study with a sample size of 296 patients was sufficient to detect differences in treatment adherence and glycemic control, as it exceeded the calculated requirement of 126 participants for adequate statistical power. This suggests that the study design was sufficiently robust to identify significant effects related to medication adherence among patients with diabetes. In total, 296 patients with a confirmed T2DM diagnosis were recruited from various healthcare facilities in Thi-Qar City. The inclusion criteria included being over 18 years old, having a confirmed T2DM diagnosis, and being willing to participate in the study. Exclusion criteria included cognitive impairment or any condition that would preclude informed consent.

### 2.3. Data Collection

#### 2.3.1. General Characteristics

We collected data on the participants' demographics (age and sex), clinical characteristics (duration of diabetes and BMI), and the presence of diabetes-related complications (microvascular and macrovascular complications).

#### 2.3.2. Treatment Regimens

Information on the type of diabetes medications that the patients were taking, including oral antidiabetic medications (OAD) and insulin, was recorded.

#### 2.3.3. Adherence Assessment

Treatment adherence was measured using the Medication Adherence Assessment Scale 8 (MAAS-8). Adherence scores were categorized into three levels: good adherence, moderate adherence, and poor adherence.

#### 2.3.4. Glycemic Control

Glycemic control was evaluated by measuring the HbA1c and random blood sugar (RBS).

#### 2.3.5. Diabetes-Related Complications

The presence of microvascular and macrovascular complications was assessed using clinical evaluations and patient records.

### 2.4. Ethical Considerations

The study adhered to the ethical standards of the Declaration of Helsinki. Informed consent was obtained from all participants, and data confidentiality was maintained throughout the study.

### 2.5. Limitations

The cross-sectional design limited the ability to establish causality. Additionally, the reliance on self-reported adherence measures may have introduced a recall bias.

### 2.6. Statistical Analysis

Data analysis was performed using SPSS Version 23 to explore the factors influencing treatment adherence in patients with diabetes and their effects on glycemic control and complications. For categorical variables, Pearson's chi-square test was used to analyze associations between demographic factors (e.g., sex and obesity) and adherence levels, ensuring that the assumptions were met. Continuous variables such as age, BMI, and HbA1c were examined using Student's *t*-test to compare means between the adherence groups, confirming normality and variance assumptions. Pearson correlation coefficient was used to assess the relationships between continuous variables, ensuring normal distribution and linearity. Statistical significance was set at *p* < 0.05 for all tests. Univariate and multivariate regression analyses were conducted to identify adherence predictors while controlling for confounders such as age, diabetes duration, and medication type.

## 3. Results

The general characteristics of the patients can be seen in Table 1. Out of the 296 patients enrolled in the study, 166 (56%) were men, with mean age of 49.4 ± 13.7 years; 72 (24.3%) were obese, and the median duration of diabetes was 5 years. Their last mean HbA1c was 8.4 ± 2.0%, with 144 (48.6%) having microvascular diabetic complications and 54 (18.2) showing macrovascular complications. Most patients were on OAD, with 74 (25.0%) participants also taking insulin. According to the MAAS-8 scoring system, 10 patients (3.4%) had good adherence scores, 90 (30.4%) showed moderate adherence, and 196 (66.2%) had poor adherence.  Table 2 explores factors that may influence adherence. Men were more likely to have poor adherence than women, with 120 (72.3%) versus 76 (58.5%), respectively (*p* = 0.003). A longer disease duration was associated with better adherence (16.8 ± 3.4 years for good adherence vs. 7.9 ± 0.6 for poor adherence, *p* = 0.010). Surprisingly, higher BMI was associated with good adherence (33.6 ± 13.5 for good adherence vs. 27.2 ± 6.1 for poor adherence, *p* = 0.009). Age, family history of diabetes or hypertension, and smoking status had no impact on the degree of adherence.

The lack of adherence–complication association may reflect time lag: Complications develop over years; our cohort's median disease duration (5 years) may be too short. Confounders: Genetics, comorbidities (e.g., hypertension), or therapies (e.g., SGLT2i) may mask adherence's role. Measurement limits: Cross-sectional design and self-reported adherence may not capture long-term effects.

**Figure 1:**
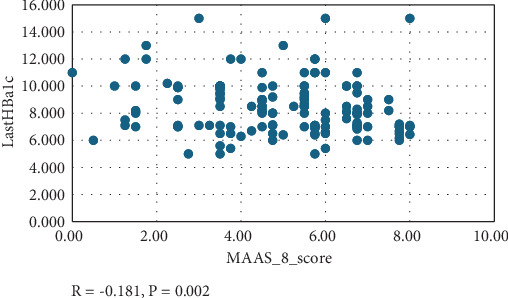
Correlation between adherence and HbA1c. *R* = −0.181, *p* = 0.002.

**Figure 2:**
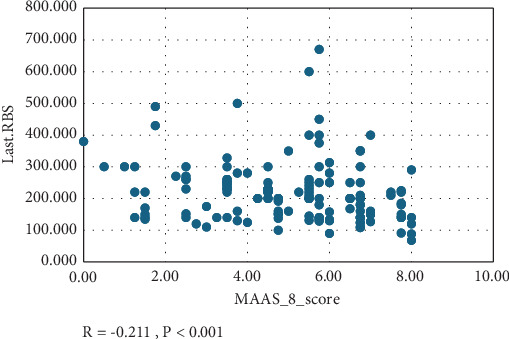
Correlation between adherence and RBS. *R* = −0.211, *p* < 0.001.

The effect of adherence on glycemic control is clearly demonstrated in both the last HbA1c and last RBS of the patients, as shown in Table 3. Among the patients with HbA1c ≥ 7%, 148 (70.5%) exhibited poor adherence, compared to 4 (40.0%) with good adherence. Conversely, for patients with HbA1c < 7%, only 48 (24.5%) displayed poor adherence versus 4 (40.0%) with good adherence (*p* = 0.014). A statistical correlation can be observed in Figure 1, where the mean RBS in milligram/deciliter was higher in the poor adherence group than in the good adherence group (237 ± 100 vs. 141 ± 83, *p* < 0.001). Notably, no patient with good adherence had an RBS ≥ 300 mg/dL (*p* = 0.078).  Figure 2 illustrates the linear correlation between adherence score and RBS. Surprisingly, there was no significant association between adherence levels and diabetes complications, including both micro- and macrovascular. However, numerical data convey that only microvascular complications increased, occurring in 40.0% of patients with good adherence compared to 44.9% with poor adherence (*p* = 0.110).

The use of different antidiabetic modalities had no effect on adherence (Table 4). However, the use of sulfonylureas (SUs) was associated with poor adherence (85.0% for SU users vs. 63.3% for non-SU users, *p* = 0.010), while patients using SGLT2i showed good adherence (16.7% for SGLT2i users vs. 2.8% for non-SGLT2i users, *p* = 0.029). Other antidiabetic treatment options seemed to have no impact on the degree of adherence.

**Figure 3:**
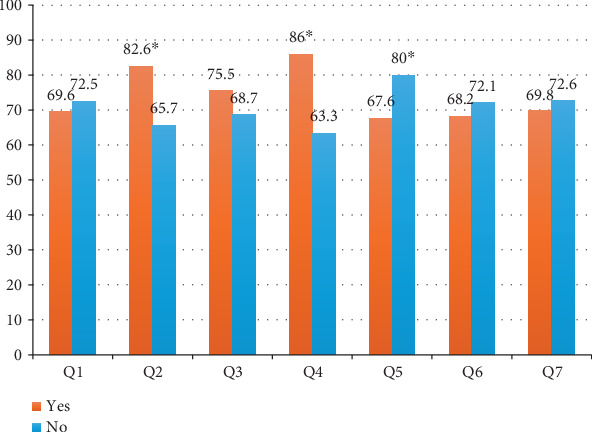
Effect of each item in MAAS-8 adherence score on the frequency of patients with HbA1c ≥ 7% (Item 8 is not a dichotomous question).

To assess the impact of each item in the MAAS-8 adherence score on glycemic control, Figure 3 shows that recent nonadherence in the last 2 weeks or during travel led to a higher percentage of patients with HbA1c ≥ 7% (82.6% and 86.0%, respectively, *p* = 0.003 and < 0.001). Surprisingly, of the 216 (73%) patients who missed at least one treatment the day before enrolment, the majority had HbA1c ≥ 7% (80.0%, *p* = 0.037).

Despite this, recent nonadherence seemed to have no such impact on the development of microvascular complications, as shown in Table 5. Contrarily, one can see a lower frequency of these complications, possibly because these complications take time to develop and recent loss of glycemic control cannot be attributed to their development. Patients who found it difficult to adhere to their medications (Q7) were more likely to develop microvascular complications (54.7% vs. 40.3%, *p* = 0.015). This factor seemed to be the only one associated with an increased risk of macrovascular complications (18.6% vs. 9.7%, *p* = 0.033).

## 4. Discussion

The aim of our study was to investigate medication adherence among 296 patients with diabetes and its impact on glycemic control and the development of complications. The general characteristics of the study participants, including demographics, clinical profiles, and treatment modalities, are summarized in Table 1.

In terms of sex distribution, our findings aligned with those of previous studies, as 56% of the participants were men and 44% were women. A previous study by Hussain et al. [7] conducted in Iraq also reported a male predominance in the prevalence of diabetes. Understanding potential sex differences in medication adherence can help tailor interventions and support strategies for optimal diabetes management.

The mean age of our study participants was 49.4 ± 13.7 years, which is consistent with the age range commonly associated with diabetes onset and prevalence. Age can significantly influence medication adherence as older individuals often face challenges such as comorbidities and polypharmacy, which may impact their treatment adherence. This finding is in line with studies by Rawnaq et al. [8] and Fadheel et al. [9], who reported similar age ranges in their respective samples.

Regarding obesity prevalence, our study revealed that 24.3% of patients were classified as obese. This finding is consistent with the global trend of increasing obesity rates and the associated risk of T2DM. Obesity is an established risk factor for the development and progression of T2DM, as it contributes to insulin resistance and impaired glucose metabolism. The high prevalence of obesity in our study population highlights the importance of incorporating lifestyle modifications, such as healthy eating habits and regular physical activity, as part of diabetes management strategies [10].

The median duration of diabetes among study participants was 5 years, indicating that most patients had been living with T2DM for a considerable period, which may influence medication adherence and disease management [11]. Previous studies have reported that a longer disease duration can impact treatment adherence due to factors such as treatment fatigue or the development of comorbidities. Understanding the relationship between disease duration and medication adherence is essential for tailoring interventions and support strategies to meet the unique needs of individuals at different stages of their diabetes journey [12].

Glycemic control was suboptimal in our study population, with a mean HbA1c level of 8.4 ± 2.0%, indicating poor blood glucose control among a significant proportion of the patients [13]. Poor glycemic control is associated with an increased risk of diabetes-related complications, including microvascular and macrovascular complications [13]. In our study, 48.6% of the patients had microvascular complications, whereas 18.2% had macrovascular complications. These findings underscore the need for improved medication adherence and effective interventions targeting glycemic control to reduce the burden of diabetes-related complications in this population.

In terms of treatment modalities, most of our patients were on OAD, whereas 25.0% were on a combination of insulin and OAD. This treatment pattern aligns with the recommendations for T2DM management, where a stepwise approach is often followed [14]. However, adherence to prescribed medications is crucial for achieving optimal treatment outcomes and avoiding complications. The association between poor medication adherence and poor glycemic control has been consistently reported in literature [15].

Interestingly, our study highlighted the effect of different antidiabetic medications on adherence patterns. Patients on SU were more likely to have poor adherence, whereas those on SGLT2i demonstrated better adherence, aligning with prior research findings [16]. Further research is needed to explore the underlying reasons for these associations and identify strategies to improve adherence in patients receiving different medication regimens.

Our study identified low levels of adherence, with only 3.4% of patients demonstrating good adherence, while the majority (66.2%) exhibited poor adherence. This finding is consistent with prior research and suggests that low adherence remains a significant concern in diabetes management [17]. An influence of sex on adherence was also observed, with men being more likely to have poor adherence than women. The impacts of disease duration, family history of diabetes, hypertension, and smoking status on patient adherence were not significant. However, the relationship between medication adherence and glycemic control was confirmed in our study, as patients with poor adherence had higher HbA1c levels and RBS measurements.

The absence of a significant adherence–complication link may stem from the study's cross-sectional design, insufficient disease duration to observe complications, or confounding effects of protective therapies. Longitudinal studies are needed to clarify long-term adherence impacts.

The elevated rates of poor adherence observed in men could be linked to cultural norms that prioritize resilience and traditional socioeconomic roles, which may conflict with self-care behaviors [9].

The findings of this study emphasize actionable strategies for healthcare providers in Thi-Qar to improve diabetes treatment adherence and glycemic control. Routine adherence screening using the MMAS-8 tool can help identify high-risk groups, such as men and SU users, for targeted interventions. Simplifying regimens by prioritizing once-daily medications like SGLT2 inhibitors may enhance adherence. Gender-sensitive interventions, such as workplace wellness programs, can address cultural barriers for men. Low-cost SMS reminders can mitigate forgetfulness, a key adherence barrier identified in the study. Aggressive monitoring of HbA1c and RBS levels in poorly adherent patients is crucial to preempt complications, even though adherence was not statistically linked to complication prevalence in this cohort. These strategies collectively aim to optimize glycemic control and reduce diabetes-related complications.

## 5. Conclusion

In conclusion, our study provides valuable insights into medication adherence among patients with diabetes, its impact on glycemic control, and the development of complications. These findings highlight the need for tailored interventions and support strategies to improve patient adherence and diabetes management. Further research is necessary to explore the complex relationship among medication adherence, glycemic control, and the development of complications.

## 6. Limitations of the Study

This study has several limitations. The cross-sectional design limits causal inference, making it impossible to establish temporal sequences (e.g., whether poor adherence leads to elevated HbA1c or vice versa) or bidirectional relationships. Self-reported adherence via the MMAS-8 is prone to recall and social desirability bias, which could be mitigated by using objective measures like electronic monitoring. Additionally, unmeasured confounding factors such as socioeconomic status, health literacy, and cultural beliefs may influence both adherence and outcomes. Finally, recruitment from a single center in Thi-Qar restricts the generalizability of findings to broader populations. Future research should include longitudinal studies with multicenter recruitment, objective adherence metrics, and comprehensive covariates to address these limitations and clarify causal pathways.

## Figures and Tables

**Table 1:** General characteristics of the patients (*n* = 296).

Age (years)	Mean ± SD	49.4 ± 13.7

Gender	Men	166 (56.1%)
	Women	130 (43.9%)

BMI (kg/m^2^)	Mean ± SD	27.3 ± 6.6
	Obese	72 (24.3%)
	Nonobese	224 (75.7%)

Duration of DM (years)	Median (quartile)	5.0 (3–10.8)

Fx of DM	*N* (%)	176 (59.5%)

HT	*N* (%)	126 (42.6%)

Smoker	*N* (%)	70 (23.6)

Last HbA1c%	Mean ± SD	8.4 ± 2.0

Last RBS (mg/dL)	Mean ± SD	220 ± 95

Diabetes-related complications		

Microvascular	*N* (%)	144 (48.6%)

Macrovascular	*N* (%)	54 (18.2%)

Number of drugs	Median (range)	2 (0–7)

On insulin	*N* (%)	74 (25.0%)

Adherence (MAAS-8)	Good	10 (3.4%)
	Moderate	90 (30.4%)
	Poor	196 (66.2%)

*Note:* MAAS-8 (good: 8, moderate: 6–< 8, poor: < 6). HbA1c, glycated hemoglobin.

Abbreviations: BMI, body mass index; DM, diabetes mellitus; Fx, family history; HT, hypertension; MAAS-8, 8-item Morisky Medication Adherence Scale; RBS, random blood sugar; SD, standard deviation.

**Table 2:** Factors associated with poor adherence.

		**Adherence**			**95% CI**		**p** ** value**
		**Good**	**Moderate**	**Poor**	**Upper**	**Lower**	
Age (years)	Mean ± SD	45.2 ± 18.8	50.2 ± 13.3	49.3 ± 13.6	47.8	50.9	0.533

Gender	Men	8 (4.8)	38 (22.9)	120 (72.3)	2.5	2.7	**0.003**
	Women	2 (1.5)	52 (40.0)	76 (58.5)	2.4	2.6	

BMI (kg/m^2^)	Mean ± SD	33.6 ± 13.5	27.0 ± 6.2	27.2 ± 6.1			**0.009**
	Obese	4 (5.6)	22 (30.6)	46 (63.9)	2.5	2.7	0.493
	Nonobese	6 (2.7)	68 (30.4)	150 (67.0)	2.4	2.7	

Duration of DM (years)	Mean ± SE	16.8 ± 3.4	8.9 ± 0.9	7.9 ± 0.6	2.4	3.03	**0.010**

Fx of DM	Yes	6 (3.4)	52 (29.5)	118 (67.0)	2.5	2.7	0.927
	No	4 (3.3)	38 (31.7)	78 (65.0)	2.5	2.7	

HT	Yes	6 (4.8)	36 (28.6)	84 (66.7)	2.5	2.7	0.474
	No	4 (2.4)	54 (31.8)	112 (65.9)	2.5	2.7	

Smoker	Yes	4 (5.7)	14 (20.0)	52 (74.3)	2.5	2.8	0.059
	No	6 (2.7)	76 (33.6)	144 (63.7)	2.5	2.6	

*Note:* MAAS-8 (good: 8, moderate: 6–< 8, poor: < 6). Data are expressed either as mean ± SD or as number (percent). Bold entries denote significant results.

Abbreviations: BMI, body mass index; DM, diabetes mellitus; Fx, family history; HT, hypertension; MAAS-8, 8-item Morisky Medication Adherence Scale; SD, standard deviation; SE, standard error of mean.

**Table 3:** Effect of adherence on glycemic control and diabetes complications.

**Variables**		**Adherence**			**95% CI**		**p** ** value**
		**Good**	**Moderate**	**Poor**	**Upper**	**Lower**	
HbA1c%	Mean ± SD	8.5 ± 3.4	7.9 ± 1.7	8.7 ± 2.0			0.016
	≥ 7	4 (40.0)	58 (64.4)	148 (75.5)	2.6	2.7	0.014
	< 7	6 (60.0)	32 (35.6)	48 (24.5)	2.3	2.6	

RBS (mg/dL)	Mean ± SD	141 ± 83	189 ± 71	237 ± 100			< 0.001
	≥ 300	0 (0.0)	10 (11.1)	38 (19.4)	2.6	2.9	0.078
	< 300	10 (100.0)	80 (88.9)	158 (80.6)	2.5	2.6	

Microvascular	Yes	4 (40.0)	52 (57.8)	88 (44.9)	2.4	2.6	0.110
	No	6 (60.0)	38 (42.2)	108 (55.1)	2.5	2.7	

Macrovascular	Yes	2 (20.0)	18 (20.0)	34 (17.3)	2.4	2.6	0.855
	No	8 (80.0)	72 (80.0)	162 (82.7)	2.5	2.7	

Any complication	Yes	6 (60.0)	54 (60.0)	94 (48.0)	2.4	2.6	0.146
	No	4 (40.0)	36 (40.0)	102 (52.0)	2.6	2.7	

Micro and macrovascular	Yes	0 (0.0)	16 (17.8)	28 (14.3)	2.4	2.7	0.301
	No	10 (100)	74 (82.2)	168 (85.7)	2.5	2.6	

*Note:* Data are expressed either as mean ± SD or as number (percent). HbA1c, glycated hemoglobin.

Abbreviations: RBS, random blood sugar; SD, standard deviation.

**Table 4:** Effect of diabetes treatments on adherence.

**Medicine**		**Adherence**			**p** ** value**
		**Good**	**Moderate**	**Poor**	
Metformin	Yes	6 (3.3)	50 (27.5)	126 (69.2)	0.369
	No	4 (3.5)	40 (35.1)	70 (61.4)	
SU	Yes	2 (5.0)	4 (10.0)	34 (85.0)	**0.010**
	No	8 (3.1)	86 (33.6)	162 (63.3)	
DPP4i	Yes	2 (10.0)	6 (30.0)	12 (60.0)	0.233
	No	8 (2.9)	84 (30.4)	184 (66.7)	
TZD	Yes	0 (0.0)	2 (25.0)	6 (75.0)	0.797
	No	10 (3.5)	88 (30.6)	190 (66.2)	
SGLT2i	Yes	2 (16.7)	4 (33.3)	6 (50.0)	**0.029**
	No	8 (2.8)	86 (30.3)	190 (66.9)	
AGIs	Yes	0 (0.0)	0 (0.0)	10 (100.0)	0.071
	No	10 (3.4)	90 (31.5)	186 (65.0)	
Insulin	Yes	2 (2.7)	24 (32.4)	48 (64.9)	0.863
	No	8 (3.6)	66 (29.7)	148 (66.7)	
GLP1RA	Yes	0 (0.0)	0 (0.0)	4 (100.0)	0.355
	No	10 (3.4)	90 (30.8)	192 (65.8)	

*Note:* MAAS-8 (good: 8, moderate: 6–< 8, poor: < 6). Data are expressed as number (percent). Bold entries denote significant results.

Abbreviations: AGIs, alpha glucosidase inhibitors; DDP4i, dipeptidyl peptidase 4 inhibitors; GLP1RA, glucagon-like peptide 1 receptor agonist; MAAS-8, 8-item Morisky Medication Adherence Scale; SGLT2i, sodium–glucose cotransporter 2 inhibitors; SU, sulfonylurea; TZD, thiazolidinedione.

**Table 5:** Effect of each item in the MAAS-8 adherence score on DM complications.

**Variables**		**Microvascular**	**Macrovascular**	**Micro and macrovascular**	**Any complication**
Q1	Yes	78 (49.4)	28 (17.7)	26 (16.5)	80 (50.6)
	No	66 (47.8)	26 (18.8)	18 (13.0)	74 (53.6)
	*p* value	0.791	0.804	0.410	0.607

Q2	Yes	36 (39.1)	16 (17.4)	14 (15.2)	38 (41.3)
	No	108 (52.9)	38 (18.6)	30 (14.7)	116 (56.9)
	*p* value	**0.028**	0.799	0.909	**0.013**

Q3	Yes	44 (44.9)	20 (20.4)	18 (18.4)	46 (46.9)
	No	100 (50.5)	34 (17.2)	26 (13.1)	108 (54.5)
	*p* value	0.364	0.497	0.233	0.218

Q4	Yes	50 (50.0)	22 (22.0)	16 (16.0)	56 (56.0)
	No	94 (48.0)	32 (16.3)	28 (14.2)	98 (50.0)
	*p* value	0.740	0.232	0.695	0.328

Q5	Yes	118 (54.6)	42 (19.4)	34 (15.7)	126 (58.3)
	No	26 (32.5)	12 (15.0)	10 (12.5)	28 (35.0)
	*p* value	**0.001**	0.379	0.486	**< 0.001**

Q6	Yes	48 (54.5)	24 (27.3)	16 (18.2)	56 (63.6)
	No	96 (46.2)	30 (14.4)	28 (13.5)	98 (47.1)
	*p* value	0.187	**0.009**	0.297	**0.009**

Q7	Yes	94 (54.7)	34 (19.8)	32 (18.6)	96 (55.8)
	No	50 (40.3)	20 (16.1)	12 (9.7)	58 (46.8)
	*p* value	**0.015**	0.424	**0.033**	0.125

*Note:* MAAS-8 (Item 8 is not a dichotomous question). Data are expressed as number (percent). Bold entries denote significant results.

Abbreviation: MAAS-8, 8-item Morisky Medication Adherence Scale.

## Data Availability

The datasets used and/or analyzed in the present study are available from the corresponding author upon reasonable request.
